# A Voice App Design for Heart Failure Self-management: Proof-of-Concept Implementation Study

**DOI:** 10.2196/40021

**Published:** 2022-12-21

**Authors:** Antonia Barbaric, Cosmin Munteanu, Heather Ross, Joseph A Cafazzo

**Affiliations:** 1 Centre for Digital Therapeutics Techna Institute University Health Network Toronto, ON Canada; 2 Institute of Health Policy, Management and Evaluation Dalla Lana School of Public Health University of Toronto Toronto, ON Canada; 3 Institute of Biomedical Engineering University of Toronto Toronto, ON Canada; 4 Institute for Communication, Culture, Information, and Technology University of Toronto Mississauga, ON Canada; 5 Technologies for Aging Gracefully Lab University of Toronto Toronto, ON Canada; 6 Ted Rogers Centre for Heart Research University Health Network Toronto, ON Canada; 7 Department of Medicine University of Toronto Toronto, ON Canada; 8 Peter Munk Cardiac Centre University Health Network Toronto, ON Canada; 9 Department of Computer Science University of Toronto Toronto, ON Canada; 10 Healthcare Human Factors Techna Institute University of Toronto Toronto, ON Canada

**Keywords:** heart failure, self-management, digital therapeutics, voice-activated technology, smart speaker, formative evaluation, mobile phone

## Abstract

**Background:**

Voice user interfaces are becoming more prevalent in health care and are commonly being used for patient engagement. There is a growing interest in identifying the potential this form of interface has on patient engagement with digital therapeutics (DTx) in chronic disease management. Making DTx accessible through an alternative interaction model also has the potential to better meet the needs of some patients, such as older adults and those with physical and cognitive impairments, based on existing research.

**Objective:**

This study aimed to evaluate how participants with heart failure interacted with a voice app version of a DTx, *Medly*, through a proof-of-concept implementation study design. The objective was to understand whether the voice app would enable the participants to successfully interact with the DTx, with a focus on acceptability and feasibility.

**Methods:**

A mixed methods concurrent triangulation design was used to better understand the acceptability and feasibility of the use of the *Medly* voice app with the study participants (N=20) over a 4-week period. Quantitative data included engagement levels, accuracy rates, and questionnaires, which were analyzed using descriptive statistics. Qualitative data included semistructured interviews and were analyzed using a qualitative descriptive approach.

**Results:**

The overall average engagement level was 73% (SD 9.5%), with a 14% decline between results of weeks 1 and 4. The biggest difference was between the average engagement levels of the oldest and youngest demographics, 84% and 43%, respectively, but these results were not significant—Kruskal-Wallis test, H(2)=3.8 (*P*=.14). The *Medly* voice app had an overall accuracy rate of 97.8% and was successful in sending data to the clinic. From an acceptability perspective, the voice app was ranked in the 80th percentile, and overall, the users felt that the voice app was not a lot of work (average of 2.1 on a 7-point Likert scale). However, the overall average score for whether users would use it in the future declined by 13%. Thematic analysis revealed the following: the theme feasibility of clinical integration had 2 subthemes, namely users adapted to the voice app’s conversational style and device unreliability, and the theme voice app acceptability had 3 subthemes, namely the device integrated well within household and users’ lives, users blamed themselves when problems arose with the voice app, and voice app was missing specific, desirable user features.

**Conclusions:**

In conclusion, participants were largely successful in using the *Medly* voice app despite some of the barriers faced, proving that an app such as this could be feasible to be deployed in the clinic. Our data begin to piece together the patient profile this technology may be most suitable for, namely those who are older, have flexible schedules, are confident in using technology, and are experiencing other medical conditions.

## Introduction

### Background

Chronic diseases are the leading cause of death and disability worldwide, with >41 million people dying every year owing to these diseases [1]. Cardiovascular diseases, such as myocardial infarctions and high blood pressure, are responsible for most chronic disease–related deaths (17.9 million people) [1]. Patient self-care is considered essential in the prevention and management of chronic diseases [2], as studies have shown the benefits of this approach, which include improved health outcomes, decreased clinic visits, and decreased health costs [3]. Mobile health is a type of digital health technology that involves the use of mobile devices for medical and public health practices [4] and enables the integration of self-care support into a patient’s routine [5]. Mobile health apps are one of the most popular tools for helping patients with chronic conditions manage their health at home [6]. However, the use of conversational agents for health-related purposes is an emerging field of research [7], and early evidence suggests that they may also be effective for the self-management of chronic diseases [8].

Conversational agents are a type of dialogue in the field of human-computer interaction and can either be voice based or typing based [9]. With voice user interfaces (VUIs), users can interact with a computing system using only speech. An example of VUIs is voice apps. The primary advantage of implementing VUIs in any environment is simplicity because it does not require the user to interact with a hand-held technology, as we are typically accustomed to. Some examples of how VUIs are being used in a clinical setting include improving physician note transcription, supporting patient registration processes, improving patient engagement with chronic disease management programs, and aging in place [10]. In a home setting, voice apps are designed to help patients manage their chronic conditions independently [11-15] and most often include informational and assistive services such as general educational content, reminders, and tracking tools. The research disseminated so far has limited efficacy in supporting final conclusions because the studies are still in development and piloting phases. As a result, there is a growing interest in investigating the feasibility of using voice apps to encourage patient engagement, specifically for chronic disease management.

### Heart Failure

Previous research has begun to investigate the feasibility of voice-activated technology for monitoring patients with heart failure (HF) [16]. HF is a cardiovascular disease that develops when the heart muscle becomes damaged or weak [17], making it difficult to pump enough blood to meet the body’s needs [18]. When this happens, fluid builds up in various parts of the body (such as the legs and ankles), creates congestion in the lungs, and leads to a lack of oxygen being delivered to the rest of the body [19]. The 2 most common causes of HF are high blood pressure and coronary artery disease; other risk factors include obesity, smoking, high cholesterol, and previous health conditions (past myocardial infarctions and heart defects at birth) [20]. It is estimated that 64.3 million people are living with HF worldwide [21].

To date, there have been limited studies investigating the potential of using a voice app for HF self-management. Some voice apps include basic functionality to help patients manage their conditions, such as asking preappointment clinical screening questions, scheduling appointments, and setting medication reminders [11,12]. Other, more recent studies have investigated using voice apps to monitor patients’ conditions through a series of symptom questions related to HF [13,16]. Feasibility was an outcome that all the studies investigated, and the results concluded that it is worthwhile to investigate how this technology can be used as an alternative platform to manage HF.

### Medly

*Medly* is an evidence-based, HF self-management program that was developed by the University Health Network (UHN) and is implemented as part of the standard of care at UHN’s Ted Rogers Center of Excellence for Heart Failure clinic [22]. The program is deployed as a mobile app, and patients access it daily using their mobile phones to log clinically relevant physiological measurements (weight, blood pressure, and heart rate) and HF-related symptoms. All patients input the same measurements and are asked the same symptom-related questions despite the stage of their HF.

The *Medly* algorithm generates an automated self-care message for the patient based on the data inputted and the patient’s medical history (determined when the patient is onboarded to the *Medly* program). The *Medly* program was deployed as a voice app as part of a previous work, and a usability study was performed with the voice app at the UHN’s Heart Failure Clinic [23].

### Objectives

The purpose of the previous usability study was mainly focused on whether the *Medly* voice app functioned as intended; feedback on the voice app design and data regarding user experience were collected. Given that the usability study took place in a controlled laboratory setting and focused on the voice app design, we sought to perform a proof-of-concept implementation study in the intended environment. The *Medly* voice app was used as a case study to investigate the broader application of voice apps for chronic disease management. The goal of this study was to determine whether voice apps can be a practical alternative for enabling patients to receive a digital therapeutic. A total of 2 constructs from the implementation framework (acceptability and feasibility) by Proctor et al [24] guided our research question: What is the acceptability and feasibility of a voice application for patients, through the use of a smart speaker, for a home chronic disease management platform? If the study findings concluded that the voice app is acceptable to patients and feasible to be deployed in a real-world setting, the inclusion of this technology to deploy digital therapeutics could add benefit to the current models of care by offering patients multiple ways to interact with these types of programs.

## Methods

### Participant Recruitment

This study asked patients with HF to interact with the *Medly* voice app in their homes for a 4-week period. The *Medly* voice app was accessed through an Amazon Alexa (Amazon.com, Inc) device; each participant was provided a device to use for the study duration. The participants were considered eligible if they had been diagnosed with HF by a physician at the UHN’s HF clinic and were prescribed the *Medly* program. The participants were also required to speak and read English adequately to understand the voice prompts in the *Medly* app. The *Medly* nurse coordinator first provided a brief overview of the research study to interested patients before introducing them to the study coordinator. If they agreed to participate, written informed consent was obtained by the study coordinator before onboarding.

Given that this study was designed as a proof of concept, a small sample size was used to gather preliminary evidence that provided insights into the success of this intervention. A total of 20 participants were recruited for the study based on similar guidance provided for pilot studies [25]. Of the 20 participants, 7 (35%) were recently onboarded (within the last 2 months) to the *Medly* program at the time when the study was being conducted. The *Medly* nurse coordinator recommended a cutoff of 2 months, given their experience with how long it typically takes for patients to settle in comfortably with the app.

All the participants were required to perform a double entry of their *Medly* measurements for the 4-week duration; more specifically, they were asked to first input their *Medly* measurements on the smartphone app before interacting with the voice app. Each participant received a gift card to compensate for their time participating in the study.

### Ethics Approval

Ethics approval was obtained from the UHN Research Ethics Board (20-6095).

### Study Outcome Measures

The evaluation of the *Medly* voice app was influenced by the implementation outcomes framework of Proctor et al [24] by focusing on 2 outcomes, specifically, acceptability and feasibility. Acceptability is defined as the perception among patients that the *Medly* voice app is agreeable or satisfactory, and feasibility is described as the extent to which the *Medly* voice app can be successfully used by patients.

### Data Collection

Data were gathered through 3 questionnaires, namely System Usability Scale (SUS) [26], National Aeronautics and Space Administration (NASA)-Task Load Index (TLX) [27], and Unified Theory of Acceptance and Use of Technology 2 (UTAUT2) [28], and semistructured interviews. Information regarding how often the voice app misheard and incorrectly recorded data was retrieved from the voice app server. During the interviews, the participants were asked about their overall experience and satisfaction with using the voice app. Other quantitative data were also collected: engagement levels (defined as the number of days the user inputted their data using the voice app divided by the total study duration—28 days) and accuracy rates (calculated by comparing the measurements inputted on the smartphone app with those recorded on the voice app). The following data were used to deduce whether the voice app was deemed acceptable by users: engagement levels, SUS, and semistructured interviews; similarly, feasibility was identified through the following: engagement levels, accuracy rates, NASA-TLX, UTAUT2 (through an effort expectancy lens), and semistructured interviews.

The study coordinator performed an onboarding session over the phone with each participant to help them set up and access the *Medly* voice app and provided them with an instruction manual (Multimedia Appendix 1). The participants were then asked a few questions regarding how comfortable they were using technology to help the study coordinator understand their comfort levels with technology (Multimedia Appendix 2). As the *Medly* smartphone app is part of the standard of care at UHN, the participants were made aware that they needed to perform a double entry of their *Medly* measurements for the 4-week duration and were told to prioritize the *Medly* smartphone app, namely to input measurements on the phone first and to follow guidance only from the smartphone app. Semistructured interviews were conducted at the end of weeks 1 and 4 and took place with the study coordinator over the phone. Questionnaires were sent out electronically at the end of weeks 2 and 4 so that the participants had privacy and felt comfortable sharing their honest thoughts and opinions.

### Study Analysis and Statistical Tests

A mixed methods, triangulation convergence model was used to draw conclusions [29]. Descriptive statistics for the standardized questionnaire responses were calculated and recorded using Microsoft Excel. Graphical representations of engagement levels were also created using Microsoft Excel. The responses from the SUS questionnaire were analyzed as per standard protocol [26], and averages were calculated for the NASA-TLX and UTAUT2 questionnaires, both overall and question specific. Data were categorized in different ways using various attributes (age, whether they were recently onboarded to the *Medly* program, whether they had any prior experience using a smart speaker, and comfort level with technology). Given the data characteristics, nonparametric statistical tests were conducted with each attribute (treated as an independent variable) for engagement levels and scores from SUS, NASA-TLX, and UTAUT2; a *P* value of <.05 was used to indicate statistical significance.

For the qualitative data, interview transcripts were analyzed and coded by the study coordinator (AB). Themes from the interviews were identified using an inductive, qualitative descriptive approach [30]. Once these themes were generated, a deductive approach was used to categorize them under the guidance of implementation outcomes framework (with a focus on the acceptability and feasibility constructs) by Proctor et al [24]. The transcripts and coding were organized using Microsoft Word. Owing to the small sample size and lack of power and statistical significance in the results, more emphasis was placed on the qualitative analysis, whereas the quantitative data and interpretations were used to support the qualitative findings.

## Results

### Characteristics of Study Participants

A total of 20 patients were recruited for the study, with a fairly even split between sexes (female: 9/20, 45%; male: 11/20, 55%) and an average age of 57.8 (SD 13.1) years. None of the participants were aged <20 years, 10% (2/20) of users were aged between 21 and 40 years, 35% (7/20) were aged between 41 and 60 years, and 55% (11/20) were aged between 61 and 80 years.

All the recruited patients were required to be enrolled in the *Medly* program, with a mix of recent onboards (7/20, 35%) and those who had been enrolled in the program for longer (13/20, 65%). The participants were also asked about their comfort levels with technology and whether they had used a smart speaker before, and 90% (18/20) of users provided responses. Regarding comfort levels, of the 18 patients, 1 (6%) patient was very uncomfortable, 0 (0%) were somewhat uncomfortable, 6 (33%) were neutral, 2 (11%) were somewhat comfortable, and 9 (50%) were very comfortable. Regarding prior use of a smart speaker, of the 18 patients, 7 (39%) indicated that they had interacted with a smart speaker before, whereas the remainder (n=11, 61%) had not.

### Quantitative Data

#### Engagement Levels and Accuracy Rates

The overall engagement level for the entire study population during the 4-week period was 73%, with noticeable drops in engagement as the weeks progressed (Table 1) and an overall decline of 14% when comparing the average engagement levels of weeks 1 and 4.

**Table 1:** Average engagement levels over the 4-week study duration.

Week	Engagement level^a^ (%), average (SD)	Days missed, average (SD)
1	80.7 (11.3)	1.4 (0.11)
2	75.0 (5.8)	1.8 (0.06)
3	70.7 (7.9)	2.0 (0.08)
4	67.1 (8.1)	2.3 (0.08)

^a^Overall average engagement level is 73.4% (SD.9.5%).

Over the 4-week duration (28 days), 9 entries (out of 411) were incorrect measurements submitted using the *Medly* voice app, indicating an overall accuracy rate of 97.8%. The errors varied between weight and blood pressure measurements. A subset (4/20, 20%) of participants was not able to successfully submit their correct readings, which led to the 9 errors that were recorded.

In addition to calculating the overall engagement levels, descriptive statistics were calculated, and the attributes mentioned previously were used to compare the results among the subgroups in the study population. The results are shown in Multimedia Appendix 3. Although some trends were identified, the statistical tests indicated no significant differences between the groups.

There was no difference in the average engagement levels between the recently onboarded (n2) and existing *Medly* patients (n1; Mann-Whitney *U*=45, n1=13, n2=7; *P*=.99). Similar to the findings related to the entire study population, engagement levels were lower in the fourth week than in the first week for both groups. Average engagement levels increased as the age groups increased, with the oldest demographic (aged 61-80 years) having the best engagement level of 84.1%, approximately double the overall engagement level of the youngest age group in the study—Kruskal-Wallis test, H(2)=3.8 (*P*=.14). Those aged 61 to 80 years were the most consistent throughout the 4-week duration and had the smallest difference among the weekly average engagement levels.

A similar trend was observed when comparing participants based on their described comfort levels with technology (statistical test results were not significant). Those who were very confident consistently used the technology more through the 4 weeks than those who reported less confidence, with a 13.6% overall difference (Mann-Whitney *U*=23.5, n1=6, n2=12; *P*=.72). There were also consistently higher engagement levels in the group that had never interacted with smart speakers before than in the group that had, with a 7.6% difference (Mann-Whitney *U*=38, n1=6, n2=12; *P*=.86). Both groups steadily declined in engagement as the weeks progressed, with similar overall differences between averages of weeks 1 and 4.

#### Acceptability of the Medly Voice App

Findings from the SUS questionnaire paired with those from the semistructured interviews were used to better understand the acceptability of using the voice app version of the *Medly* program.

The responses from the SUS questionnaire from the second week resulted in an overall average score of 69 (out of 100), ranking the voice app in the 53rd percentile based on previous studies. By contrast, the average score from the fourth week was 77 (out of 100), ranking it in the 80th percentile based on previous studies. These data indicated an overall increase in the level of satisfaction with using the *Medly* voice app (by 27%) in the study population. The difference in the averages for each individual question between weeks 2 and 4 was also calculated, with the last question in the survey having the biggest difference of 13%. The participants felt that as time went on, they needed to learn more things about the voice app to successfully interact with it (consistent with the NASA-TLX cognitive load results). Response distributions in the results of weeks 2 and 4 were fairly similar for all the questions (Figure S1 in Multimedia Appendix 4).

Average SUS scores were also calculated based on the different patient characteristics (age, *Medly* status, comfort levels, and familiarity with interacting with a smart speaker). Overall, the scores were similar in range for all the characteristics. However, the largest range in the data was identified in the age groups, with the oldest (61-80 age group) demographic providing the lowest score (72 out of 100), ranking it in the 62nd percentile, whereas the middle-aged demographic provided an average score of 87.5 (out of 100), ranking it in the 96th percentile. The average score from the youngest demographic was 77.5, ranking it in the 80th percentile. The Kruskal-Wallis test showed that these findings had no significant difference—H(2)=0.89 (*P*=.64).

#### Feasibility of the Medly Voice App

The NASA-TLX questionnaire was used in this study to better assess the workload perceived by the study participants when using the *Medly* voice app. A 4% increase was seen in the average scores between the results of weeks 2 and 4, indicating a slightly higher workload. Although the averages for each of the questions were fairly low, questions relating to (1) success rates; (2) how hard they needed to work to accomplish the task; and (3) feelings of discouragement, irritation, and stress scored worse than the rest of the questions. The results are shown in Figure S2 in Multimedia Appendix 4. The participants also felt less successful with using the *Medly* voice app at the end of the study than they did at the end of week 2 (22% difference in the results).

When analyzing the scores based on the different age groups, it was found that the youngest demographic felt that they needed to work the most (highest average of 2.67) when compared with the middle-aged (average of 1.61) and oldest demographics (average of 2.12); the results of the Kruskal-Wallis test was not insignificant—H(2)=0.039 (*P*=.98). It was also found that those who were newly onboarded to the *Medly* program felt more rushed when using the voice app and less successful when inputting their measurements as compared with those who had been on the *Medly* program for a longer time (approximately 15% difference in scores for each question); Mann-Whitney test was not significant (*U*=25.5, n1=12, n2=6; *P*=.73). The difference in the average scores for those who described themselves as less confident when using technology consistently gave poorer scores for each of the questions, indicating that they had a more difficult time than those who described themselves as confident; the Mann-Whitney test was also not significant (*U*=11, n1=12, n2=6; *P*=.55; Table S1 in Multimedia Appendix 4).

In summary, the descriptive statistics showed that the youngest age group felt that they needed to work the most, the study population collectively felt that they needed to put in slightly more effort as time went on, and those who were less familiar with technology had more difficulty using the voice app than those who were more confident.

The UTAUT2 questionnaire was used to better understand participants’ thoughts regarding facilitating conditions, effort expectancy, habit, and behavioral intention when it came to using the voice app. The biggest difference between the results of weeks 2 and 4 was regarding whether they would use the *Medly* voice app in the future, with a 13% decline in the average score. The oldest demographic was the least keen on using it in the future, whereas the middle-aged demographic was the most interested in future use; the Kruskal-Wallis test indicated these results to be not statistically significant—H(2)=1.88 (*P*=.39). When asked whether the voice app became a habit, those who had used the technology before agreed more than those who had not (19% difference in the responses), although this test was also not statistically significant (Mann-Whitney *U*=38, n1=7, n2=13; *P*=.86).

Overall, all the participants felt that the voice app required low effort to use and that it was easy for them to operate. They were less certain about whether using the voice app had become a habit for them (this can be supported by engagement levels) and were least certain about whether they would use the voice app in the future, as shown in Table S2 in Multimedia Appendix 4.

### Qualitative Data

The interview themes were classified using implementation outcomes by Proctor et al [24], specifically focusing on the *feasibility* and *acceptability* constructs to answer the research question. The themes (1) feasibility of clinical integration and (2) voice app acceptability are presented in the subsequent sections, each with their own set of accompanying subthemes.

#### Feasibility of Clinical Integration

The feasibility of clinical integration was influenced by several factors; in our findings, the 2 subthemes of (1) users adapting to the voice app’s conversational style and (2) device unreliability helped determine the potential that this technology has to be integrated into existing workflows and practice. Whether the users are able to adapt to the voice app and the extent to which the device is considered unreliable will identify the feasibility of the voice app being realistically used in the clinical environment. Further details regarding these 2 subthemes are provided in the subsequent sections.

#### Users Adapting to the Voice App’s Conversational Style

Most participants found the device setup and instructions fairly straightforward but at times struggled to successfully log their measurements on the *Medly* voice app. When the participants struggled, they adjusted the way they spoke instead of continuing in their natural manner in hope that the voice app would understand them better:

I learned how to get into her rhythm as opposed to her getting into my rhythm.

Participant 04

Specific strategies were used to change their speaking style, which most often involved modifying the volume, tone, pace, and style of their speech. Different strategies seemed to work better for different participants, specifically with the pace at which they spoke:

Now I just say 116.4 pounds (faster) and there’s absolutely no issues with her now.

Participant 12

Of course I would either make sure to be speaking directly at it or elevate my voice or something like that.

Participant 15

I want to record one hundred, but it’s very typical to say “a hundred” and not “one hundred,” but I notice it doesn’t pick up on that.

Participant 17

Once the participants changed their conversational tone when speaking to the voice app, they began to notice difficulties in the interaction because it no longer felt like a natural conversation:

It’s like when you talk to someone foreign or you know from another country or another language and you try to say a few words for them to understand it.

Participant 12

I try to, like, separate each word, almost like I had to speak robotic.

Participant 18

I have to be serious, slow and sure of how I say the numbers.

Participant 17

Another interaction strategy adopted by most participants involved using the touchscreen capabilities of the device. In most cases, this alternative input was the favorable approach over using voice because it was simpler to use and, most importantly, faster:

I got into a routine which allowed me to go through it as quickly as possible, and that routine would be that I would speak the results for weight, blood pressure and heart rate, and then I would interact directly on the touch screen for symptoms so we didn’t have to wait for her. So yes, every time I use the touch screen it works fine and the fact that I could use a touch screen and it would work even though she hadn’t finished speaking is a big plus for me.

Participant 15

Interactions were found to be most successful when the participants did not multitask on other items:

You can multitask if you really want to, but that’s what I think mistakes can be made easier.

Participant 02

I knew the questions that were going to be asked after a while, but I still listened. Only because you know I’d rather do it right than wrong if I can.

Participant 08

Despite the learning curve experienced by most participants, the mitigation strategies described earlier support the feasibility of deploying a voice app, such as *Medly*, in the clinic because of the perseverance displayed by these participants to make the interaction easier for themselves over time.

#### Device Unreliability

Almost all the participants experienced some level of difficulty when they interacted with the voice app. Sometimes, the voice app froze, and the session ended abruptly; at other times, it would not provide the user with an opportunity to correct any of the wrong measurements:

You can go back and correct it, right, but sometimes it gives you a little bit of a hassle so I have to start over.

Participant 02

Then she just shut down...When she couldn’t get the measurements or something, she would just turn off.

Participant 04

The participants also described instances where the voice app was unable to correctly pick up the information they were saying, making them feel frustrated, annoyed, panicked, and discouraged to the point where they no longer wanted to use the device that day:

Yeah, I’d wake up in a great mood and oftentimes it was so frustrating that it made me cranky afterwards. Yeah, it really switched my mood. One time she repeated it to me and I thought she got it alright and then she repeated it and said that I fainted and I had not fainted, so I panicked.

Participant 18

When the voice app was unable to pick up the correct measurements, the participants often felt the need to speak louder. This was considered to be problematic specifically in situations where a participant may not be feeling well and does not have the ability to project their voice. As explained by one of the participants, with the smartphone, they were able to share information without needing to exert a lot of energy:

I would never want it to not be on my phone when I go into the hospital and I have a hard time talking. If my blood pressure is through the roof or it’s way too low from retaining water, it’s so hard to speak and I love that I could just throw my phone at the doctor and be like “look, this was [my data] two days ago”...I really like that.

Participant 18

Although the voice app seemed feasible to deploy from a patient interaction perspective, the users also experienced difficulties when interacting with the device for various tech-related reasons. Understanding the causes and frequencies of these malfunctions will help identify when and where it is appropriate to use voice apps such as *Medly*.

#### Voice App Acceptability

This theme described the extent to which the study participants found the *Medly* voice app satisfactory. This level of acceptability included not only the participants’ thoughts but also other factors that may have influenced their experience, as described by the following subthemes: (1) the device integrated well within household and users’ lives, (2) the users blamed themselves when problems arose with the voice app, and (3) the voice app was missing specific features desired by the users.

#### Device Integration in the Household

In addition to using the device to access the *Medly* voice app, many participants also found that they used it for other purposes during their time in the study. Over the 4 weeks, some participants described the device as a companion, with one of the participants noting the following:

She became like a buddy. I know it’s little quirks, specifically when it makes mistakes...I would say for people that live on their own or whatever it can become like a friend, right?

Participant 08

Some participants also described their experience interacting with the device as “pleasant,” and others specifically felt the need to use manners and be polite while conversing with it:

And I’ve gotten along with Alexa just fine. It was so cute. I was inputting on Medly and I did it with Alexa at the same time and at the end I said “Alexa, thank you” and she said “you bet”...One night I said, “oh Alexa goodnight” and she said “night night, sleep well.”

Participant 08

The device became a companion not only for the users but also for their family members and friends:

She did give my granddaughter a knock knock joke the other night. [The grandkids] have fun with her by asking what the weather is or something like that.

Participant 10

This interaction is an example of how easily the device can fit in and become integrated within a space in the household. While in common areas, the users have noted using the device for other activities, such as the following:

I let it play music for me or I ask what’s the weather like today and I do the CTV News first thing in the morning, so yeah, I think it’s a great thing.

Participant 02

Having the device in common spaces also served as a reminder for some participants who had difficulty remembering to perform their *Medly* measurements. Others also mentioned that because the device was placed in a common space, they would be more inclined to use *Medly* on it:

Seeing the monitor right there on the counter I feel like it definitely encourages and motivates me and is a visual reminder as opposed to the app on the phone to actually do it.

Participant 11

At first I thought it would be my phone. But probably you know, now it’s Alexa. She sits right there, so probably Alexa.

Participant 02

Some participants also placed the device in other places in their house, such as the bedroom. In these cases as well, they found the setup useful:

I use it at night time when I’m going to bed like you know, relaxing music.

Participant 06

Furthermore, in some cases, the voice app was more preferred when compared with the smartphone:

I’m in my bedroom and I have a bathroom in the room, so when I go in the bathroom to weigh myself, I do my blood pressure at the same time. So ideally that is where I talk to [Alexa]...over the last week it’s been working and I really like that because then I’m done and then I can go right back to bed after I take my pills so it doesn’t make my mind wake up.

Participant 03

I’m sort of having concussion symptoms and the phone makes me nauseous. So at the moment, I prefer only having to do it with Alexa.

Participant 14

Despite the benefits of the device integrating well within different spaces in the household, there are drawbacks that can exist when keeping the device in a public space. Most participants noted the importance of having a quiet space to focus and successfully submit their readings:

Honestly like I did it more often when I didn’t have my son because everything here he likes to speak over me...He would repeat ‘Alexa’ behind me.

Participant 18

Like if my husband would walk into the kitchen as I was doing it, I would shoo him away, literally.

Participant 08

#### Users Blamed Themselves When Problems Arose With the Voice App

Although some participants experienced frustration when the device abruptly stopped working or incorrectly heard them, often times (especially in the first week), the users felt that it was their fault when a mistake happened:

I wasn’t annoyed by it. I just thought, oh, I’m not speaking clearly or loudly enough, or you know.

Participant 08

Well again, I go back to the learning curve in the first week. There was some frustration, but you can’t blame that on Alexa, that was all me.

Participant 05

These reflections indicated that the users were generally understanding of the voice app and had some patience when interacting with it.

#### Missing but Desired Voice App Features

The participants shared some of the features they valued in devices that programs such as *Medly* can be offered on. In particular, the users preferred to interact with a device that is fast and can quickly record their data for the day. In some instances, the users compared the capability of the voice app with that of Bluetooth, indicating that Bluetooth is a much faster and simpler process:

It’s just really cumbersome, like the whole process. And I guess part of that is because the [smartphone] app is so easy. And I think it could get even easier if I got the Bluetooth blood pressure and scale.

Participant 04

To me, honestly, because they want it in the morning, the smartphone is much faster.

Participant 09

Most users also expressed concern about how they would use the voice app should they go on an overnight trip. A device that is small enough to be portable when traveling was desired and often mentioned:

The only thing I don’t like about it is it is big and bulky so it is not something I would be too inclined to want to travel with. So yeah, so for me the mobility issue would be a bit of a concern if I had to rely on it.

Participant 13

## Discussion

### Principal Findings

This manuscript presents the findings from a proof-of-concept implementation study for a voice app designed for patients with HF using a mixed methods approach. To our knowledge, this is the first evaluation of a voice app used for helping patients manage an advanced chronic condition at home. To date, studies have only reported on accuracy and acceptability levels in a controlled laboratory environment; however, these findings are still consistent with the results presented in this paper [11,14]. Although the SUS scores were higher in week 4 than in week 2, engagement levels declined by 14% between the start and end of the study. The participants felt that they needed to use a higher cognitive load in week 4 than in week 2 (4% increase), and the average rating regarding whether they would use it in the future decreased by 13%. An accuracy rate of 97.8% indicates that the participants were able to successfully log their measurements most of the time, which may have led to the higher SUS score. Some qualitative findings can be potential reasons why engagement levels declined. In particular, from a feasibility perspective, the device was at times unreliable, and the users had to work (to varying efforts) to adapt to the flow of the conversation. Although this may have been tolerable in the first few weeks, over time, it may have become tiresome, depending on how quickly the users adapted. Similarly, because the users often blamed themselves when mistakes arose, this could have created a negative association with the voice app, and over time, the users may have begun to feel discouraged from using it.

To better understand the voice app’s acceptability and feasibility of implementation, we sought to identify any noticeable differences between the participants in terms of engagement levels. Although our quantitative data are not statistically significant, our observed findings are similar to those presented by Ware et al [31], namely the finding that engagement levels were highest in the older age group and progressively lower in the younger age groups. This finding is also consistent with other research that specifically focused on the use of voice-based conversational agents among the older adult population [4,32-37]. Although the oldest group had the highest engagement levels, the middle-aged demographic (aged 41-60 years) had the highest average SUS score, indicating that they were the most accepting of the voice app. Although we cannot conclude any findings definitely based on these observations, it provides a starting point for future work.

One of the most common responses provided by the participants during interviews was the notion that the voice app takes a long time to complete and, in particular, takes longer than the *Medly* smartphone app. The users often described being rushed out the door in the mornings, in which case they appreciated being able to use the smartphone app to quickly input their measurements. This type of lifestyle and response was observed less with the older demographics, who generally seemed to have more patience and understanding when interacting with the voice app. There were also specific cases in which the voice app actually proved to be more useful than the smartphone. One of the participants was experiencing concussion-type symptoms and, as a result, had limited screen time, so the voice app worked well for them. Another participant often felt fatigue as one of the side effects of their medications and experienced difficulties navigating the *Medly* smartphone app in the mornings. In this case, they also appreciated how much easier it was to perform the required tasks using the *Medly* voice app. Similar sentiments were echoed by other participants who realized that they can successfully record their readings when speaking in a relaxed, nonstrenuous manner. Although this worked well for some participants, one of the participants in a similar situation had a different experience, specifically because the voice app was unable to decipher their speech when they were feeling unwell owing to their weak and fragile voice. As a result, further advancements are required to better recognize sound, specifically when users are unable to exert large amounts of energy while speaking. Similar technical limitations have also been outlined in other studies on voice apps [15].

The findings from this study also show how well integrated the device became in many households and the potential benefits this may have for participants. Owing to the versatility of the device, it quickly became a part of many users’ daily routines, from listening to music to asking for dinner recipes, and even started turning into a companion. Not only did the device provide social support, but it also served as a visual reminder to perform their *Medly* measurements. A participant noted that they would be more inclined to use the *Medly* voice app simply because it was in a common space they frequent in their house. Therefore, the natural integration of the device into users’ lives over the 4 weeks shows the possibility that it may make it more convenient for some to perform their *Medly* measurements and may encourage and motivate others who often forget.

These findings help begin to uncover the “profile” of the patient demographic this technology would be most suitable for. We suspect that those who are older adults (aged >60 years), feel more confident in using technology, and have less busy schedules have an easier time, are more successful, and are consistent when interacting with the voice app. In addition, those with multimorbidity can benefit from using this platform, especially because of the common side effects they may experience from their conditions.

### Comparison With Prior Work

To our knowledge, this study is part of only a few studies that have investigated the use of a voice app for a chronic disease in the intended environment for a prolonged period (4-week duration). Similarly, this work is one of the firsts to study a voice app that is designed to be personalized to individual patients (output responses depend on the parameters set when the patient is onboarded to the program). A systematic review performed by Bérubé et al [38] specifically focused on voice-based conversational agents for chronic health conditions and found only 2 voice apps designed as conversational agents for HF [39,40]. Both studies were primarily focused on the system architecture and accuracy of speech recognition, and one of the studies relied on the smartphone to implement the voice-based assistant. Other studies have focused on the acceptance and feasibility of voice apps for HF through preliminary assessments, such as survey responses based on usability studies performed in controlled environments [11,12]; the results of all these studies showed the promise that this technology has in the field of chronic disease management, especially for HF. Finally, 2 more recent studies investigated the engagement [13] and feasibility [16] of an HF-related voice app for a longer duration (90 days). The study performed by Apergi et al [13] showed higher engagement with the older patient cohort (similar to this study’s results), and Shara et al [16] reported favorable perception and high comfort levels in their study population. This study begins to uncover the potential that a voice app platform has for a program, such as *Medly*, and provides a basis for future work to explore who may benefit the most from this platform and why.

### Limitations

Multiple limitations were identified over the course of the study and, as a result, should be acknowledged to better understand the impact of the findings.

First, because there were numerous questionnaires and interviews, the study team was mindful of the potential for social desirability bias [41]. As a result, the participants were encouraged to speak honestly and were given the opportunity to disclose their thoughts through questionnaires privately instead of over the phone. Second, because this study was a proof of concept for a voice app in its intended environment, the sample size was not statistically powered, and most of the findings were interpreted in a qualitative manner. Future work should design studies with statistical significance (including using a validated questionnaire to capture user comfort levels with technology) to better understand who this may be most beneficial for. Third, specific study factors could have impacted the participant’s thoughts, experiences, and feedback. The users were aware that the study duration was only a 4-week period and, as a result, may have had higher engagement levels than if they were asked to use the voice app for a longer period. The participants were also required to perform a double entry of their measurements; the study results may have differed if users were only required to use the voice app. Fourth, because the inclusion criteria were general enough to include any patient enrolled in the program, selection bias likely occurred during recruitment. In this case, there may have been missed opportunities to include a greater variety of demographics in the study, especially those who primarily spoke languages other than English. Finally, because most participants in this study had never interacted with a smart speaker before, their thoughts and feedback may have been influenced by the fact that they were interacting with a novel technology. As a result, their thoughts on the device itself could be reflected in their responses, even though any VUI device could have been used in the study.

### Conclusions

This study used a mixed methods approach to investigate the acceptability and feasibility of deploying a voice app for digital therapeutics used in chronic disease management. Overall, our findings conclude that the participants were largely successful in using the *Medly* voice app despite some of the barriers faced, proving that an app such as this could be feasible to be deployed in the clinic for future use. Our data begin to piece together the patient profile that this technology may be most suitable for. Future work should involve a statistically powered study that investigates the following demographics: those who are older (>60 years), have less busy schedules, exhibit high confidence levels when using technology, or experience symptoms (such as fatigue or headaches) from chronic conditions.

## Appendix

Multimedia Appendix 1 Instruction manual for the *Medly* voice app.

Multimedia Appendix 2 Baseline questionnaire for the participants.

Multimedia Appendix 3 Overall and weekly average engagement levels based on various patient characteristics.

Multimedia Appendix 4 Data showcasing the positive (a) and negative (b) attribute question and results from the System Usability Scale questionnaire, with week 2 data on top and week 4 data on the bottom (Figure S1). National Aeronautics and Space Administration (NASA)-Task Load Index score distributions in the results of weeks 2 and 4 (top and bottom, respectively; Figure S2). Average scores for each NASA-Task Load Index question (Table S1). Average scores for each of the constructs from the Unified Theory of Acceptance and Use of Technology 2 questionnaire (Table S2).

## Abbreviations

HF: heart failure
NASA: National Aeronautics and Space Administration
SUS: System Usability Scale
TLX: Task Load Index
UHN: University Health Network
UTAUT2: Unified Theory of Acceptance and Use of Technology 2
VUI: voice user interface
