# Supplementary material for: A Voice App Design for Heart Failure Self-management: Proof-of-Concept Implementation Study
Source: JMIR Form Res. 2022 Dec 21;6(12):e40021. doi: 10.2196/40021 (PMC9813814; doi:10.2196/40021)
Supplement: Multimedia Appendix 1 [file formative_v6i12e40021_app1.docx]

# Multimedia Appendix 1


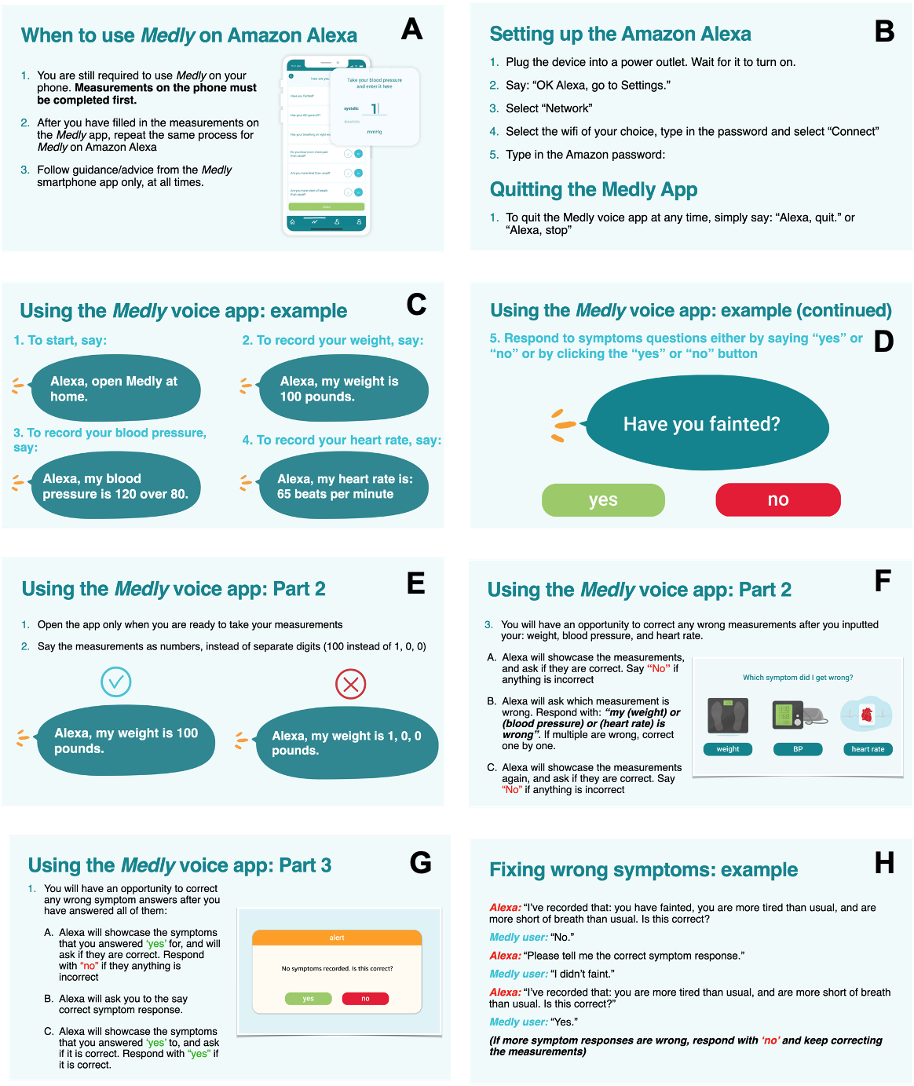


**Fig S1. Instructions manual for *Medly* voice app.** Example pages taken from the instructions manual that is given to participants when using the Medly voice app. Figures produced and designed by Jacqueline Simpson. (A) describes the importance of first interacting with the Medly smartphone app and only taking directions from it, since the Medly voice app is part of a clinical trial, (B) explaining to users how to set up the Amazon device to access the Medly voice app, (C) and (D) showcasing an example of the conversation that may occur in the first half of the interaction, (E), (F) and (G) providing more details explaining the instructions on how to properly use the device and how to correct any mistakes, (H) showcasing how to correct any wrong symptom responses that may have been recorded
