# Supplementary material for: A Voice App Design for Heart Failure Self-management: Proof-of-Concept Implementation Study
Source: JMIR Form Res. 2022 Dec 21;6(12):e40021. doi: 10.2196/40021 (PMC9813814; doi:10.2196/40021)
Supplement: Multimedia Appendix 2 [file formative_v6i12e40021_app2.docx]

# Multimedia Appendix 2

**Table S1. Baseline questionnaire for participants.**

| *Question* | *Response* |
| --- | --- |
| On a scale of 1-5, how comfortable are you with using technology (a smartphone, the computer, a tablet)? Please explain. (See below for a scale description).    *Scale description:*  *1: Need help from a family member when using these types of devices. Can’t do it by myself.*  *5: Feel confident in my ability and never need any help. Can easily troubleshoot and fix problems on my own mostly.* |  |
| Have you ever used or interacted with an Amazon Alexa or Google Home before? Approximately how many times? (never, once a week, twice a week, every day, once a month, only once at a friend’s house etc.) |  |
| How do you feel about using an Amazon Alexa device over the next 30 days? Are you: scared, confident, concerned, excited, worried, or neutral? Why? |  |
| Do you have any worries or concerns about using an Amazon Alexa device over the next 30 days? |  |
| Do you have any difficulties using a smartphone device? Is there anything that you dislike when you use your phone? |  |
