# Supplementary material for: A Voice App Design for Heart Failure Self-management: Proof-of-Concept Implementation Study
Source: JMIR Form Res. 2022 Dec 21;6(12):e40021. doi: 10.2196/40021 (PMC9813814; doi:10.2196/40021)
Supplement: Multimedia Appendix 3 [file formative_v6i12e40021_app3.docx]

# Multimedia Appendix 3

**Table S1. Overall and weekly average engagement levels based on various patient characteristics.**

| **Patient Characteristic** | **Overall** | **Week 1** | **Week 2** | **Week 3** | **Week 4** |
| --- | --- | --- | --- | --- | --- |
| *Medly status (%)* | | | | | |
| **New** | *72.5* | 75.5 | 71.4 | 75.5 | 67.4 |
| **Existing** | *73.9* | 83.5 | 76.9 | 68.1 | 67.0 |
| Age (%) | | | | | |
| **20 - 40** | *42.9* | 57.1 | 42.9 | 35.7 | 35.7 |
| **41 - 60** | *75.6* | 90.5 | 81.0 | 71.4 | 59.5 |
| **61 - 80** | *84.1* | 85.7 | 84.4 | 83.1 | 83.1 |
| *Comfort level with technology (%)* | | | | | |
| **Levels 1 - 3 (very uncomfortable, somewhat uncomfortable, neutral)** | *70.8* | 83.3 | 78.6 | 66.7 | 54.8 |
| **Levels 4 - 5 (somewhat comfortable, very comfortable)** | *84.4* | 88.3 | 83.1 | 81.8 | 84.4 |
| *Interaction with smart speaker before (%)* | | | | | |
| **Yes** | *76.0* | 85.7 | 85.5 | 71.4 | 71.4 |
| **No** | *83.6* | 88.1 | 86.9 | 82.1 | 77.4 |
