# Supplementary material for: A Voice App Design for Heart Failure Self-management: Proof-of-Concept Implementation Study
Source: JMIR Form Res. 2022 Dec 21;6(12):e40021. doi: 10.2196/40021 (PMC9813814; doi:10.2196/40021)
Supplement: Multimedia Appendix 4 [file formative_v6i12e40021_app4.docx]

# Multimedia Appendix 4


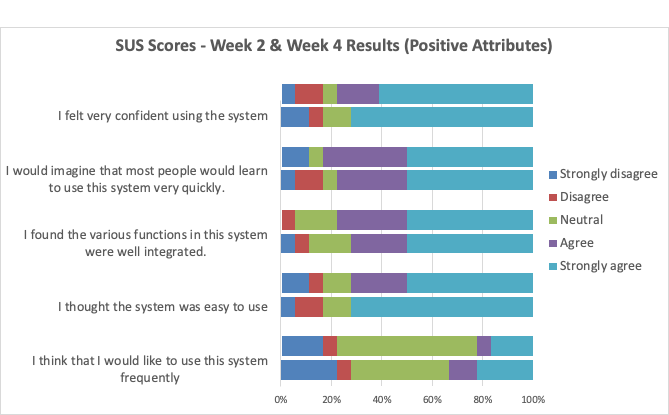


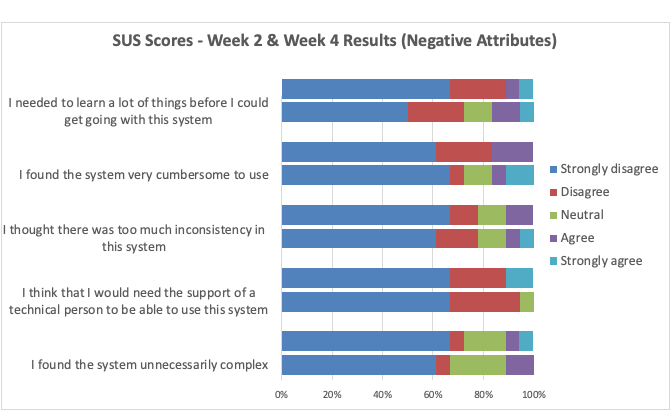


(b)

**Fig S1.** Data showcasing the positive (a) and negative (b) attribute question and results from the SUS questionnaire, with week two data on top and week four data on the bottom.


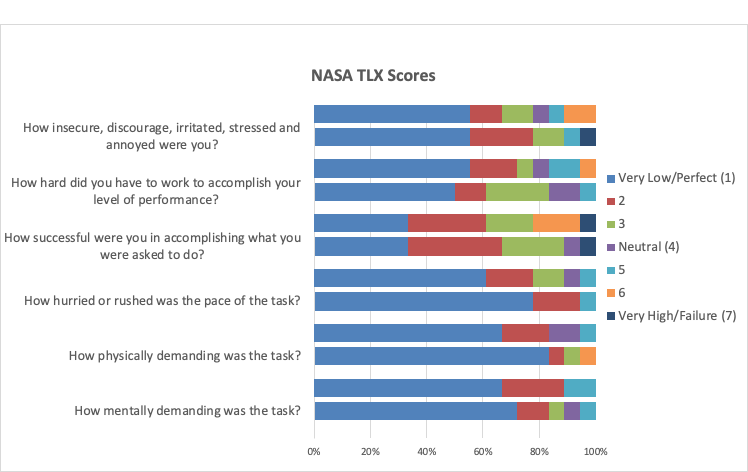


**Fig S2.** National Aeronautics and Space Administration (NASA)-Task Load Index score distributions in the results of weeks 2 and 4 (top and bottom, respectively).

**Table S1. Average scores for each NASA-Task Load Index question.**

| **Average** | **Mental demand** | **Physical demand** | **Feelings of being hurried or rushed** | **Success rate** | **Level of difficulty to accomplish task** | **Feelings of insecurity, discouragement, irritation, and/or stress** |
| --- | --- | --- | --- | --- | --- | --- |
| Comfort level: 1 - 3 | 1.78 | 1.67 | 2.00 | 2.78 | 2.44 | 2.11 |
| Comfort level: 4 - 5 | 1.11 | 1.44 | 1.44 | 2.33 | 1.56 | 2.00 |
| Difference between CF 1-3 and CF 4-5 | 9.5% | 3.2% | 7.9% | 6.3% | 12.7% | 1.6% |

**Table S2. Average scores for each of the constructs from the Unified Theory of Acceptance and Use of Technology 2 questionnaire.**

|  | Effort expectancy | Facilitating conditions | Habit | Behavioral intention |
| --- | --- | --- | --- | --- |
| Overall average (out of 7) | 5.9 | 5.9 | 4.8 | 4.5 |
